# Assessment of pharmacological activities of two medicinal plant of Bangladesh: *Launaea sarmentosa* and *Aegialitis rotundifolia roxb* in the management of pain, pyrexia and inflammation

**DOI:** 10.1186/0717-6287-47-55

**Published:** 2014-10-28

**Authors:** Golam Sarwar Raju, Md Mizanur Rahman Moghal, Mohammad Salim Hossain, Md Mahadi Hassan, Md Mustahsan Billah, Sayed Koushik Ahamed, SM Masud Rana

**Affiliations:** Department of Pharmacy, Noakhali Science and Technology University, Sonapur, Noakhali, 3814 Bangladesh; Department of Pharmacy, Dhaka International University, Dhaka, Bangladesh

**Keywords:** *Launaea sarmentosa*, *Aegialitis rotundifolia roxb*, Analgesic, Antipyretic, Anti-inflammatory

## Abstract

**Background:**

The current study aims at evaluating the analgesic, anti-pyretic and anti-inflammatory properties of methanolic extract of the stem, bark and leaves of *Launaea sarmentosa* and *Aegialitis rotundifolia roxb.*

**Results:**

The AELS and AEAR extract presented a significant (***p < 0.001) dose dependent increase in reaction time in writhing method and showed inhibition of 63.1% and 57.1% respectively at the doses of 400 mg/kg body weight while standard drug showed (P < 0.001) inhibition of 69.23%. In tail immersion method, AELS and AEAR showed maximum time of tail retention at 30 min in hot water i.e. 6.93 sec and 6.54 sec respectively at highest doses of 400 mg/kg body weight than lower dose while standard pentazocine showed reaction time of 7.62 sec. The AELS and AEAR extract also exhibited promising anti-inflammatory effect as demonstrated by statistically significant inhibition of paw volume by 32.48% and 26.75% respectively at the dose of 400 mg/kg body weight while the value at the dose of 200 mg/kg body weight were linear to higher dose at the 3^rd^ hour of study. On the other hand, Standard indomethacin inhibited 40.13% of inflammation (***P < 0.001). In Cotton-pellet granuloma method, AELS and AEAR extract at the dose of 400 mg/kg body weight exhibited inhibition of inflammation of 34.7% and 29.1% respectively while standard drug showed (P < 0.001) inhibition of 63.22%. Intraperitoneal administration of AELS and AEAR showed dose dependent decrease in body temperature in brewer’s yeast induced hyperthermia in rats at both doses. However, AELS significantly decreased body temperature (***p < 0.001) at 400 mg/kg compared to control.

**Conclusions:**

Present work propose that the methanolic extract of *Launaea sarmentosa* and *Aegialitis rotundifolia roxb* possesses dose dependent pharmacological action which supports its therapeutic use in folk medicine possibly mediated through the inhibition or blocking of release of prostaglandin and/or actions of vasoactive substances such as histamine, serotonin and kinins.

## Background

Inflammation in response to cellular injury is a complex physiological reaction of the body that is marked by tissue swelling, capillary dilation and anti-histamine activity result in redness, heat and pain, tumor and loss of function [1]. It is generally initiated by several factors ranging from bacterial infection and chemical injury to environmental pollution that result in apoptosis, cell injury or death [2, 3]. Tissue injury induced by this injury or trauma results in the release of inflammatory mediators including cytokines, tumor necrosis factor (TNF-α), interleukin-1 (IL-1) from leukocytes, monocytes and macrophages [4]. The cytokines further trigger the up-regulation of other pro-inflammatory cytokines, chemokines, immunoglobulins as well as increase the expression of many cellular adhesion molecules (CAMs) and their physiological action [5].

Also there is an increase in the expression of phospholipase A2, cyclooxygenase- 2 (COX-2), 5-lipoxygenase (5-LOX) and inducible nitric oxide synthase (iNOS) [6, 7] which work collectively to increase vasodilatation and permeability of blood vessels [8, 9] and leads to increased blood flow, exudation of plasma proteins and fluids and migration of leukocytes, mainly neutrophils into the injured tissues [10, 11]. For instance, the regulation of prostaglandin i.e. eicosanoid synthesis is a classic mechanism for controlling inflammation and pain [12, 13].

On the other hand, Pain is mainly a defensive mechanism of the body and is an ill-defined, unpleasant sensation and emotional experience along with acute or chronic tissue damage which is usually induced by an external or internal noxious stimuli [14, 15]. The subsequent elaboration of mediators such as interleukin-1 and tumor necrosis factor- TNF-α is believed to propagate the synthesis, release and action of autacoid prostaglandin E2 (PGE2) and F2α by the endothelium and pericytes of brain capillaries that excite pain nerve endings [16, 17]. The increase in prostaglandin levels within the peritoneal cavity increases capillary permeability and thus enhances inflammatory pain [18].

Pyrexia or fever is due to secondary impact of malignancy, inflammation, tissue damage, infections or other diseased states [19]. The infected or damaged tissue initiates the secretion and enhanced formation of pro-inflammatory chemical mediators (cytokines e.g. interleukins-1 β, α, β and TNF-α) which upsurge the synthesis of prostaglandin E2 (PgE2) near the pre-optic hypothalamic area, thereby triggering the hypothalamus activity to elevate normal body temperature [20, 21]. Most of the antipyretic drugs acts to reduce the elevated body temperature by inhibiting COX-2 expression, thus PGE2 biosynthesis [22].

Drugs or compounds which are used currently for the management of fever, pain and inflammatory conditions are either non-steroidal like aspirin or steroidal like corticosteroids. All of these drugs also possess additional toxic side effects like allergic reactions, renal failure, hearing loss or they may rise the risk of hemorrhage by affecting platelet coagulation function [23, 24]. Therefore, development of newer safe and more influential anti-inflammatory drugs with lesser side effects are necessary. Studies suggest that aromatic plant and herbs (ginger and turmeric) may block cyclooxygenase (COX) and lipoxygenase (LOX) pathways thereby inhibiting the release of prostaglandin, leukotriene and also inhibit the release of histamine [25–28].

*Launaea pinnatifida* Cass. Synonym *L. sarmentosa* (Willd.) is belong to Asteraceae family, locally known as kulhafila in the Maldives, is a creeping, perennial procumbent, stoloniferous herb, native to tropical Indian coastlines [29, 30]. The leaflets of *L. sarmentosa* are simple toothed form with an average size of 12-15 cm long. Leaves are slightly bitter in taste and have characteristic odor. The roots of *L. sarmentosa* showed the presence of lactiferous cells, pitted vessels, simple fibers, calcium oxalate crystals, devoid of pith and contains alkaloids, amino acids, glycosides, tannin, carbohydrates and steroids [29, 31]. It is used by mothers after child birth mainly in a lehya preparation known as Hilibeys. It has popularity in the treatment of abdominal disorders, urinary infections [29]. *L. sarmentosa* is also testified to possess soporific, tonic, diuretic and aperient properties and is used as a substitute for Taraxacum [32]. Whole plant is used in rheumatoid arthritis, gout and the leaf in rheumatism and also to heal skin injuries caused by fish spines while fishing [33]. Herb of these plant are fed to buffaloes as a galactagogue [34]. Another study affirmed the use of whole plant as a bath decoction to treat skin related diseases [35].

*Aegialitis rotundifolia Roxb* locally known as banrua belong to Plumbaginaceae family are small trees or woody mangrove shrubs that grow up to 2–3 meter tall and is available in the sandy or rocky soils of more saline area of Sundarbans of west Bengal and are endemic to the coastal part of South Asia [36–38]. The deciduous species have leafy stems with leathery leaves, the flowers are organized in terminal cymose racemous inflorescences forms and fruits are dehiscent and have a spongy mesocarp [39]. Locally it is pounded with oil and applied to relief insect bites pain but there is no scientific evidence to support this therapeutic use. There is no strong evidence on the use of that genus in folklore medicine and the phytochemical compounds are not so well identified.

With this background, the present study was undertaken to inspect the activity of the aforementioned traditional claims of *L. sarmentosa* and *A. rotundifolia roxb.* Thus, we investigated the analgesic, anti-pyretic and anti-inflammatory effects of proposed plant on the peripheral and central nervous systems by using several validated experimental methods in trial animals. Preliminary acute toxicity test were also carried out to evaluate the safety dose range of these widely used medicinal plant.

## Results

### Antipyretic activity test

**Figure 1:**
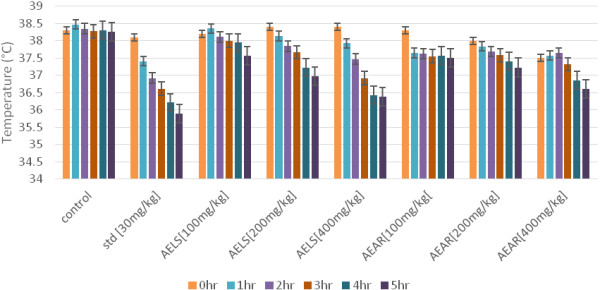
**Inhibition of pyrexia of AELS, AEAR and STD.**

The results of brewer’s yeast-induced anti-pyretic activity of AELS and AEAR were shown in Table 1. It was observed that 400 mg/kg of AELS showed maximum antipyretic activity by reducing the temp at of 36.38°C at 5^th^ hour while compared with standard acetyl salicylic acid that reduces the temp at 35.89°C. On the other hand, 400 mg/kg of AEAR showed moderate antipyretic activity by reducing the temp at 36.61°C. Figure 1 shows the rectal temperature (°C), (P < 0.001, 0.01 and 0.05) for the test and standard when compared to the control. The test drugs yields a dose dependent decrease in rectal temperature at various time intervals.

**Table 1:** **Effects of AELS, AEAR and Standard drug on brewer’s yeast induced pyrexia**

Group	Treatment	Dose [mg/kg]	Temp [t _0_ b]°C	Temp [t _0_ a]°C	Rectal temperature °C in mean ± SEM				
					1 hrs.	2 hrs.	3 hrs.	4 hrs.	5 hrs.
I	Control	---	37.26 ± 0.1	38.3 ± 0.1	38.47 ± 0.1	38.35 ± 0.2	38.28 ± 0.1	38.31 ± 0.1	38.27 ± 0.1
II	Std	250	37.21 ± 0.1	38.1 ± 0.3	37.41 ± 0.3*	36.92 ± 0.2***	36.61 ± 0.1***	36.21 ± 0.2***	35.89 ± 0.1***
III	AELS	100	37.26 ± 0.1	38.2 ± 0.2	38.36 ± 0.11*	38.11 ± 0.1*	38.0 ± 0.13*	37.95 ± 0.12*	37.56 ± 0.1*
IV	AELS	200	37.31 ± 0.3	38.4 ± 0.1	38.14 ± 0.10**	37.85 ± 0.13**	37.67 ± 0.13**	37.23 ± 0.11**	36.98 ± 0.15**
V	AELS	400	37.29 ± 0.1	38.4 ± 0.1	37.93 ± 0.09**	37.47 ± 0.08***	36.92 ± 0.10**	36.43 ± 0.11***	36.38 ± 0.09***
VI	AEAR	100	37.34 ± 0.2	38.3 ± 0.3	37.65 ± 0.10	37.63 ± 0.13	37.55 ± 0.12	37.57 ± 0.10	37.5 ± 0.11
VII	AEAR	200	37.42 ± 0.1	38.0 ± 0.1	37.84 ± 0.11*	37.69 ± 0.10*	37.58 ± 0.14*	37.41 ± 0.20*	37.23 ± 0.18*
VIII	AEAR	400	37.27 ± 0.1	37.5 ± 0.1	37.57 ± 0.17**	37.65 ± 0.15**	37.32 ± 0.13**	36.86 ± 0.11**	36.61 ± 0.18**

Results are expressed as mean ± S.E.M. t_0_b = initial body temperature prior to injection of Brewer s yeast, t_0_a = body temperature 18 h after injection of brewer s yeast, n = 6 each group, *P < 0.05, **P < 0.01, ***P < 0.001, significantly different value while compared to the control groups, (ANOVA followed by Dunnet’s t-test).

### Analgesic effect

#### Tail immersion test

**Figure 2:**
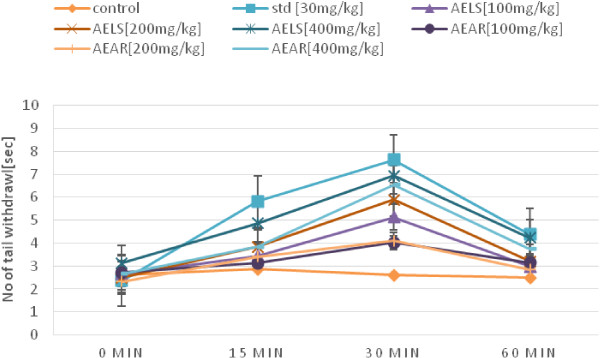
**Reaction time for tail immersion test of AELS, AEAR and STD.**

Table 2 shows the results of the tail immersion test. At a dose of 400 mg/kg body weight, AELS (***P < 0.001) and AEAR (**P < 0.01) shows significant reaction time of 6.93 sec and 6.54 sec respectively at 30 min after extract administration while standard pentazocine showed significant reaction time of 7.62 sec. In this model, Figure 2 displays that reaction time increased significantly for the test and standard when compared to the control. The test drugs yield a dose dependent increase in reaction time at various time intervals.

**Table 2:** **Effects of AELS, AEAR and pentazocine on tail-immersion test**

Group	Treatment	Dose [mg/kg]	Average tail withdrawing time (sec) (Mean ± S.E.M)			
			0 min	15 min	30 min	60 min
I	Control	---	2.62 ± 0.05	2.87 ± 0.07	2.61 ± 0.11	2.49 ± 0.018
II	Standard	30	2.36 ± 0.06**	5.81 ± 0.04***	7.62 ± 0.06***	4.38 ± 0.05***
III	AELS	100	2.66 ± 0.02*	3.44 ± 0.05*	5.13 ± 0.03*	2.98 ± 0.03*
IV	AELS	200	2.5 ± 0.01**	3.83 ± 0.05**	5.9 ± 0.04**	3.21 ± 0.04**
V	AELS	400	3.12 ± 0.02**	4.86 ± 0.01***	6.93 ± 0.05***	4.24 ± 0.03***
VI	AEAR	100	2.71 ± 0.05	3.13 ± 0.04	4.03 ± 0.05	3.15 ± 0.08
VII	AEAR	200	2.33 ± 0.02*	3.41 ± 0.03*	4.1 ± 0.07*	2.85 ± 0.01*
VIII	AEAR	400	2.67 ± 0.01**	3.84 ± 0.04**	6.54 ± 0.07**	3.74 ± 0.03**

Values are expressed as mean ± S.E.M (n = 6). *P < 0.05 (significant), **P < 0.01 (more significant) and ***P < 0.001 (most significant) compared with vehicle control (ANOVA followed by Dunnet’s t-test).

### Acetic acid induced writhing test

**Figure 3:**
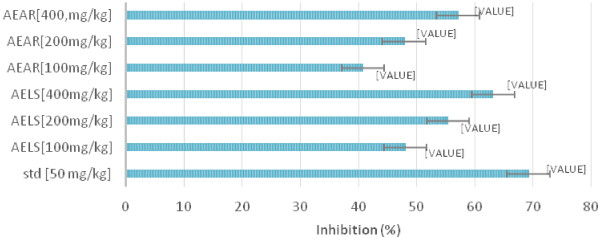
**Percent Inhibition of pain of AELS, AEAR and STD.**

Table 3 demonstrate the results of analgesic effect by acetic acid induced writhing method. At a dose of 400 mg/kg body weight, AELS shows most significant (***P < 0.001) inhibition of 63.1% while AEAR shows inhibition (**P < 0.01) of 57.1%. On the other hand, At a dose of 200 mg/kg body weight, AELS and AEAR shows inhibition of 55.36% (P < 0.01) and 47.86% (P < 0.05) respectively. Again at a dose of 100 mg/kg body weight, AELS and AEAR shows inhibition of 48.05% and 40.76% respectively (*P < 0.05) while at 50 mg/kg of standard drug showed (***P < 0.001) inhibition of 69.23%. Figure 3 shows the percent inhibition of standard, AELS and AEAR.

**Table 3:** **Effects of AELS, AEAR and aspirin on acetic acid induced writhing method**

Group	Treatment	Dose [mg/kg]	No. of writhes [15 min] (Mean ± S.E.M)	Inhibition (%)
I	Control	---	48.33 ± 1.13	---
II	Standard	50	14.87 ± 0.09***	69.23%
III	AELS	100	25.1 ± 0.12*	48.05%
IV	AELS	200	21.57 ± 0.11**	55.36%
V	AELS	400	17.83 ± 0.1***	63.10%
VI	AEAR	100	28.63 ± 0.14	40.76%
VII	AEAR	200	25.20 ± 0.13*	47.86%
VIII	AEAR	400	20.73 ± 0.10**	57.10%

Values are expressed as mean ± S.E.M (n = 6). *P < 0.05 (significant), **P < 0.01 (more significant) and ***P < 0.001 (most significant) compared with vehicle control (ANOVA followed by Dunnet’s t-test).

### Anti-inflammatory effect

#### Carrageenan induced hind paw edema model

**Figure 4:**
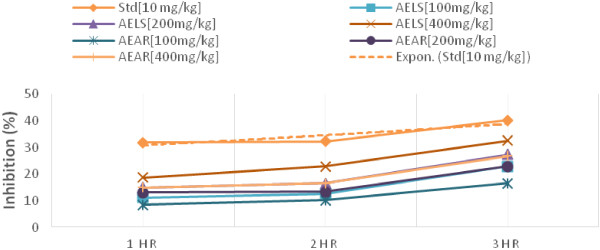
**Inhibition of inflammation of AELS, AEAR and STD.**

The effect of AELS and AEAR on carrageenan induced hind paw edema method in rats shown in Table 4. The results obtained indicates that, 400 mg/kg of AELS at 3^rd^ hours showed maximum inhibition (***P < 0.001) of 32.48% while AEAR showed (**P < 0.01) inhibition of 26.75%. Again at the dose of 200 mg/kg, AELS and AEAR showed inhibition of 27.39% (P < 0.01) and 22.93% (P < 0.05) respectively while Standard indomethacin inhibited 40.13% of inflammation (***P < 0.001) at the same time. Figure 4 shows the percent of inhibition of inflammation for the test and standard drug. The test drugs yield a dose dependent increase of inhibition at various time intervals.

**Table 4:** **Effects of AELS, AEAR and Standard drug on carrageenan induced inflammation on rats**

Group	Treatment	Dose [mg/kg]	Paw volume at different time interval (in ml) (Mean ± S.E.M)				Inhibition (%)		
			0 hour	1 hour	2 hour	3 hour	1 hour	2 hour	3 hour
I	Control	---	.77 ± .048	1.07 ± .059	1.27 ± .052	1.57 ± .011	---	---	---
II	Standard	10	.61 ± .003	.73 ± .02**	.86 ± .03***	0.94 ± .02***	31.8	32.3	40.13
III	AELS	100	.72 ± .04	.95 ± .05*	1.11 ± .05*	1.21 ± .04*	11.21	12.59	22.93
IV	AELS	200	.67 ± .03	.91 ± .03**	1.06 ± .06**	1.14 ± .005**	15	16.5	27.39
V	AELS	400	.73 ± .03	.87 ± .04**	.98 ± .04***	1.06 ± .05***	18.7	22.8	32.48
VI	AEAR	100	0.74 ± 0.02	0.98 ± .02	1.14 ± .03	1.31 ± .05	8.41	10.23	16.56
VII	AEAR	200	.69 ± .03	.93 ± .05*	1.1 ± .06*	1.21 ± .04*	13.0	13.39	22.93
VIII	AEAR	400	.64 ± .03	.91 ± .03**	1.06 ± .06**	1.15 ± .06**	15.0	16.5	26.75

Values are expressed as mean ± S.E.M (n = 6). *P < 0.05 (significant), **P < 0.01 (more significant) and ***P < 0.001 (most significant), compared with vehicle control (ANOVA followed by Dunnet’s t-test).

### Cotton-pellet granuloma test

**Figure 5:**
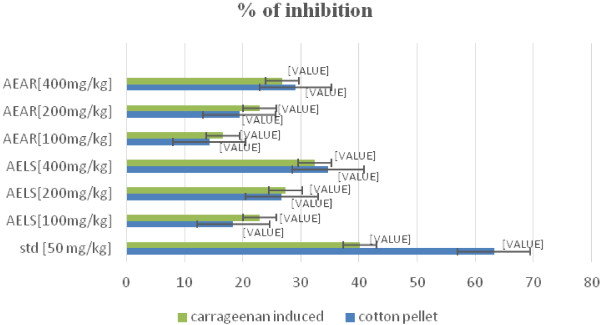
**Relation of % inhibition of inflammation in both carrageenan and cotton pellet model.**

Table 5 shows the results of anti-inflammatory effect of cotton-pellet granuloma method. At a dose of 400 mg/kg and 200 mg/kg body weight, AELS showed most significant (***P < 0.001) and significant (**P < 0.01) value respectively i.e. inhibition of 34.7% and 26.72%. On the other hand, at a dose of 400 mg/kg and 200 mg/kg body weight, AEAR showed 29.1% (P < 0.01) and 19.43% (P < 0.05) inhibition respectively while at 40 mg/kg of standard drug showed (***P < 0.001) inhibition of 63.22%. To confer the anti-inflammatory activity, Figure 5 shows the relation in the percent inhibition of inflammation of AENH, AESM and AEAN in both Carrageenan induced (at 3^rd^ hour) and Cotton-pellet granuloma test model.

**Table 5:** **Anti-inflammatory activity of AELS, AEAR and Standard drug on Cotton-pellet granuloma induced inflammation on rats**

Group	Treatment	Dose [mg/kg]	Weight of dry cotton pellet granuloma (mg)	Inhibition (%)
I	Control	---	41.12 ± 0.32	---
II	Standard	40	15.12 ± 0.09***	63.22
III	AELS	100	33.56 ± 0.21*	18.38
IV	AELS	200	30.13 ± 0.20**	26.72
V	AELS	400	26.85 ± 0.17***	34.7
VI	AEAR	100	35.26 ± 0.19	14.25
VII	AEAR	200	33.13 ± 0.20*	19.43
VIII	AEAR	400	29.15 ± 0.18**	29.1

Values are expressed as mean ± S.E. (n = 6). *P < 0.05, **P < 0.01 and ***P < 0.001 compared with vehicle control (ANOVA followed by Dunnet’s t-test).

## Discussion

Our present study was to evaluate the pharmacological effect of methanol extract of *Launaea sarmentosa* and *Aegialitis rotundifolia roxb* on animals while the acute toxicity study exposes that this plant might be considered as a broad nontoxic one. Results regarding various method showed that these plant possess a significant inhibition and reduction of pain, inflammation and pyrexia with a reasonable safety profile.

Pain and pyrexia is generally induced by tissue damage, infections, inflammation, malignancy and other disease-states or condition, thereby initiates the enhanced formation of pro-inflammatory mediators which boost body temperature [20, 21]. Drugs or compounds having antipyretic activity usually show an inhibitory effect on cyclooxygenase-2 expression, thus inhibiting biosynthesis of PGE2 and consequently decreases the elevated body temperature [40]. Yeast-induced fever or pyrexia is also called pathogenic fever and its etiology could be the production of prostaglandins which set the thermoregulatory center to increase body temperature [41]. Yeast-induced pyrexia is considered as a useful test for the screening of synthetic drugs as well as plant extracts for their antipyretic activity [41, 42]. These agents or compounds acts to suppress the peripheral formation of pyrogenic cytokines while blocking the COX pathways and lowering the thermoregulatory set point. In our present study, the intraperitoneal administration of *L. sarmentosa* in yeast induced fevered rat shows significant attenuated rectal temperature while *A. rotundifolia roxb* show moderate significant activity and their effect are comparable to that of standard anti-pyretic drug, aspirin. It is inferred that such activity of the extract may be due to the presence of active non-steroidal compounds, single bioactive substance or a mixture of compounds and inhibition of prostaglandin biosynthesis could be the possible mechanism of action of such antipyretic activity. Also, there are several other mediators or multi-processes introducing the pathogenesis of fever and inhibition of any of these responsible mediators may bring such anti-pyresis [43]. Flavonoids are common polar compounds that have previously reported in our plant extract [29, 31] which are generally known to have such anti-pyretic activity [44, 45].

Acetic acid induced writhing method is generally used to determine peripherally acting analgesic action and represents pain sensation which acts by triggering localized inflammatory reaction. This method is not only simple, reliable, sensitive but also affords rapid evaluation of peripheral analgesic action [46]. In our study, intraperitoneal administration of noxious chemical acetic acid causes the twisting of dorsoabdominal muscles constrictions because of irritation of peritoneal cavity in which acetic acid is thought to discharge of prostaglandins E2 and F2α mediators as well as lipoxygenase mediators that excite pain nerve endings by inflammatory response [47]. These endogenous mediators of inflammatory pain are sensitive to non-steroidal anti-inflammatory drugs (NSAID) and opioids [48]. It is well known that non-steroidal anti-inflammatory and analgesic drugs play role in diminishing the inflammatory pain at the peripheral target sites by inhibiting or blocking the formation of pain mediators whereas bradykinin and prostaglandins are responsible for pain process [49]. In the present study, the analgesic action of *L. sarmentosa* and *A. rotundifolia roxb* can be attributed to the significant reduction of writhes in mice, suggests that the analgesic effect may be mediated peripherally via the inhibition of release and synthesis of PGs and other endogenous mediators. It also suggests that *L. sarmentosa* has superior inhibitory action than *A. rotundifolia* roxb in the inhibition of COX pathway which is actually involved in prostaglandin biosynthesis*.*

Although acetic acid induced writhing method in animals is a very sensitive but not a selective pain test. Thus to corroborate that the plant has analgesic activity, tail immersion tests were also directed which signify the centrally acting analgesic activity. Tail immersion test is well-thought to spinal response or reflexes, but could also involve higher neural structures which mainly confers central analgesic action [50] and is also highly correlated with relief of human pain perception [51]. From the results of above two methods, it can be inferred that *L. sarmentosa* showed central and peripheral analgesic action in both tail immersion and acetic acid writhing method which is more significant than *A. rotundifolia roxb.* Preliminary phytochemical studies of *L. sarmentosa* revealed the presence of terpenoid, tannin, glycoside, alkaloid [29, 31] which are generally known to involve in the inhibition of synthesis of prostaglandins, leukotriene’s, and other endogenous substances that are key players in triggering pain perception [44, 52, 53].

Pain and inflammation are pathophysiologically related with various clinical situations such as cancer, arthritis and vascular diseases. The testing of inflammation by carrageenan-induced is designated because of its selectivity in identifying orally active inflammatory agents, particularly in the severe phase of inflammation [54]. It is treated as a significant predictive method for the screening of anti-inflammatory agents which acts to inhibit the mediators of acute inflammation [55, 56]. The carrageenan-induced paw edema in rat is a biphasic process. The histamine or serotonin is released in the first phase and the bradykinin, prostaglandin, protease, and lysosome is produced in second phase [57]. Based on these reports and obtained results, it is concluded that the methanolic extract of *L. sarmentosa* and *A. rotundifolia roxb* exhibited significant suppression of paw edema formation in rats that was measured in the third hour of experimentation and that perhaps acts by inhibiting the release and/or local actions of vasoactive substances. It is suggested based on the facts that the late phase of carrageenan-induced edema is accompanying with the release of prostaglandin-like mediators and is clinically sensitive to steroidal and non-steroidal (NSAID) anti-inflammatory agents.

On the other hand, chronic inflammation develop when the body response is insufficient to eliminate the pro-inflammatory agents. Thus proliferation of fibroblasts and the infiltration of cells, neutrophils and exudation [58, 59] occurs which by means of the early development of proliferative cells. These developed cells can either be in spread or granuloma form [59]. The cotton pellet-induced granuloma model is widely used to assess the proliferative components of chronic inflammation in where a subcutaneously imbedded cotton pellet in the rats show a transudate phase, an exudative phase and a proliferative phase of the inflammatory response [60]. Reduction in dry pellet weight could result due to decrease production of fibroblasts and synthesis of collagen and mucopolysaccharides during formation of granuloma tissue and thus signifies the suppression of inflammatory proliferative phase [61]. In the present study, administration of *L. sarmentosa* (400 mg/kg, p.o.) was found to reduce the weight of cotton pellet in a dose dependent manner and significantly reduced the granuloma tissue formation by higher doses of the extract which is close to the inhibitory effect of standard indomethacin and is better than the effect of *A. rotundifolia roxb*.

This investigation suggest that smaller doses are less effective in reducing migration of leucocytes into the areas of inflammation, since it is known that granuloma formation is due to leukocyte accumulation [62]. Flavonoids and terpenoids are compounds effective in treating acute inflammation whereas steroids and glycosides are the constituents that are effective in chronic inflammation [63]. These phytocompounds are well recognized for anti-inflammatory possessions as well as inhibition of pain perception which have previously reported in the extract of *L. sarmentosa*[29, 31]. Flavonoids are well known compound for the analgesic, anti-inflammatory and antipyretic activities [44, 45]. So it is anticipated that the present studies will stimulate further efforts towards the development of new, safe, more effective and urgently needed medications with lesser side effects for the treatment of fever, pain and inflammatory diseases.

## Conclusions

On the basis of these findings, it may be inferred that methanolic extract of *L. sarmentosa* and *A. rotundifolia roxb* has moderate analgesic, anti-pyretic and anti-inflammatory activities. It also found that, these activities were related to the dose and these results corroborate the traditional and local use of these plant in folk medicine. At present, there are no extensive reports on investigation to identify the main active phytochemical components present in methanolic extract of *L. sarmentosa* and *A. rotundifolia roxb*. Further investigations are anticipated to identify those active components present, in order to development of a potent and safe analgesic, anti-inflammatory and antipyretic drug.

## Methods

### Plant material

The bark, stem and leaves of *L. sarmentosa* and *A. rotundifolia roxb* were collected from the Sonadia deep of Cox’s Bazar. The plant material was originally identified and authenticated by Bangladesh National Herbarium, Mirpur, Dhaka. The voucher specimen numbers were DACB: 38312 and DACB: 38310 for *L. sarmentosa* and *A. rotundifolia roxb* respectively.

### Preparation of extracts

The bark, stem and leaves of *L. sarmentosa* and *A. rotundifolia roxb* were washed thrice with speedy running tape water and twice with distilled water to remove the adhered salts, soils and other associated animals, parasites and then dried in shade at temperature between 21-30°C for 15 days. After the process of drying, these were grounded in blender into fine powders. Then 450 gm of each fine powdered crude plant was subjected for cold extraction process by macerating with 2100 ml of 98% methanol at room temperature for a week. At the end of 7 days, each macerate was filtrate with whatman No 1 filter paper and evaporated using a rotatory evaporator (Bűchi 011, USA) so as to get semi-solid extract. Residue left at the bottom of the beaker is the crude methanol extract of *L. sarmentosa* and *A. rotundifolia roxb*. The filtrate finally obtained was air dried and kept in refrigerator maintained at 4°C for further test.

### Experimental animals

In the experiment, Swiss Albino Mice (25-30 g) and Wister Albino Rats (180-210 g) of either sex were used. They were collected from Jahangir Nagar University of Dhaka, Bangladesh. Animals of both category were kept in cages of polypropylene and feed on water ad libitum and standard laboratory pellet diet. Animals were uncovered to alternate cycle of 12 h light and dark while temperature of 25 ± 2°C and relative humidity of 55 ± 10% were also maintained. Animals were permitted for 7 days to adapt to the laboratory conditions before the start of experiment. This present project work was cleared by the Institutional Animal Ethical Committee [IAEC].

### Acute toxicity studies

According to the procedures set by organization for economic co-operation and development guidelines (OECD), acute toxicity studies were accomplished for the plant extracts of AELS and AEAR [64]. Swiss Albino Mice (25-30 g) and Wister Albino Rats (180-210 g) of same age and either sex were taken into account to evaluate the acute toxicity level of proposed plant extract. For the experiment they were fasted whole night and only water ad libitum was allowed. Thus, different groups having six mice in each were orally administered with different prepared aqueous extracts of 100, 200, 400, 800 and 1000 mg/kg p.o respectively. After dosing, the rate of mortality and common behavior of these groups were witnessed continuously for initial 4 h, and intermittently for 6 h and then again at 24 h and 48 h respectively. In these experiment, doses of 1000 mg/kg show significant reduction in survival of mice (LD50- around 1000 mg/kg/b.wt). Hence, 100/200/400 mg/kg/b.wt of AELS and AEAR was considered for the present studies. Common behaviors of these animals such as convulsion, pilo erection, aggressiveness, loss of lighting reflex, analgesia, grooming, skin color, sedation, hypnosis, diarrhea and respiratory rate were also observed after dose administration [65].

### Chemicals and drugs

Carragennan (Sigma Aldrich Inc., St Louis, MO, USA), Brewer’s Yeast (Arkopharma, Carros, France), Acetic acid (Merck Germany), Methanol (Merck Germany), Standard Diclofenac sodium, Indomethacin, aspirin, pentazocine and acetyl salicylic acid (Beximco Pharmaceuticals Ltd., Bangladesh). Chemicals used for present work were of analytical grade and arranged in the form of suspensions using a few drops of suspending agent diluted with distilled water.

### Methods for the evaluation of anti-pyretic effect

#### Brewer’s yeast induced pyrexia method

This model was run to study the anti-pyretic activity, slightly modifying the method described by *Adams et al.*[66]. Total 8 groups of six animals each are taken into individual cages. To bring fever, 20% aqueous suspension of Brewer’s yeast (10 ml/kg) were injected into all group of animal’s dorsum region. About 18 h after the injection of pyretic agent, the rectal temperature was measured individually by a digital thermometer (SK-1250 MC, Sato keiryoki Mfg). Only those animals that shows an increase in temperature of at least 0.6°C are certified for the experiment. At 18 hour of brewer’s yeast administration, the following doses of sample was directed.

- Group I: Treated as control, received vehicle 10 ml/kg normal saline p.o.
- Group II: Received standard drug acetyl salicylic acid (250 mg/kg p.o.) dissolved in distilled water
- Group III: Received AELS 100 mg/kg p.o. suspended in 2% w/v gum acacia solution.
- Group IV: Received AELS 200 mg/kg p.o. suspended in 2% w/v gum acacia solution.
- Group V: Received AELS 400 mg/kg p.o. suspended in 2% w/v gum acacia solution
- Group VI: Received AEAR 100 mg/kg p.o. suspended in 2% w/v gum acacia solution.
- Group VII: Received AEAR 200 mg/kg p.o. suspended in 2% w/v gum acacia solution.
- Group VIII: Received AEAR 400 mg/kg p.o. suspended in 2% w/v gum acacia solution.

After sample and drug administration, rectal temperature of each rate was recorded by a digital thermometer at 0, 1, 2 and 3 hr. Thus average rectal temperature of each group (n = 6) was calculated and compared with standard drug acetyl salicylic acid.

### Methods for the evaluation of analgesic effect

#### Tail immersion method

In the present study, analgesia was evaluated following Luiz *et al.* method [67]. Total 8 groups of six animals each are taken into individual cages and the animals were allowed to adopt in the cage environment for half an hour before the start of experiment. All animals were held in position with the tail extending out in a suitable restrainer. Freshly filled hot water maintained at 55.0 ± 1.0°C were used to immerse the lower 5 cm portion of the tail and the time to withdrawal of the tail was noted as the reaction time or tail flick latency. Any animals fail to withdraw its tail from hot water within 10 sec is rejected from the experiment.

- Group I: Control group animals received 10 ml/kg of 0.5% sodium lauryl sulphate (SLS).
- Group II: animals received Standard drug Pentazocine at dose of 30 mg/kg i.p.
- Group III: Received AELS 100 mg/kg in 0.5% SLS p.o.
- Group IV: Received AELS 200 mg/kg in 0.5% SLS p.o.
- Group V: Received AELS 400 mg/kg in 0.5% SLS p.o.
- Group VI: Received AEAR 100 mg/kg in 0.5% SLS p.o.
- Group VII: Received AEAR 200 mg/kg in 0.5% SLS p.o.
- Group VIII: Received AEAR 400 mg/kg in 0.5% SLS p.o.

After administration of above scheduled dose, the reaction time was counted at 0, 15, 30 and 60 min respectively. The mean reaction time for each group was recorded and compared with the value of standard [68].

### Acetic acid induced writhing method

Peripheral analgesic activity is widely assessed by using acetic acid induced writhing technique in which acetic acid solution was intraperitoneally injected and the number of stretching’s and writhings was counted as previously reported by Koster *et al*. and Hendershot & Forsaith *et al.*[69, 70]. Total 8 groups of six animals each are employed into individual cages.

- Group I: Control group animals received 10 ml/kg of 2% tween.
- Group II: Standard group animals received Aspirin at dose of 50 mg/kg i.p.
- Group III: Received AELS 100 mg/kg in 2% tween p.o.
- Group IV: Received AELS 200 mg/kg in 2% tween p.o.
- Group V: Received AELS 400 mg/kg in 2% tween p.o.
- Group VI: Received AEAR 100 mg/kg in 2% tween p.o.
- Group VII: Received AEAR 200 mg/kg in 2% tween p.o.
- Group VIII: Received AEAR 400 mg/kg in 2% tween p.o.

All the scheduled test doses were administered orally 1 hr prior to injection of acetic acid intraperitonealy (0.1 ml of 0.6% v/v). After the five minutes of acetic acid injection, the number of writhing movements was recorded for individual animals for a period of 15 min which was considered as withering of the abdominal muscles of animal accompanied by stretching with a jerk at the hind limb. The counted number of writhing in each treated animal group was then compared to that of a control group.

$$\begin{array}{r} \%\;\mathrm{inhibition}\;\mathrm{formula}\;=\;\left[\left(C\;‒\;T\right)\;/\;C\right]\;\times \;100\%\\ \;\mathrm{Where},\;C\;=\;\mathrm{Mean}\;\mathrm{of}\;\mathrm{control}\\ T\;=\;\mathrm{Mean}\;\mathrm{of}\;\mathrm{treated}\; \end{array}$$

### Methods for the evaluation of anti-inflammatory effect

#### Carrageenan induced hind paw edema model

Anti-inflammatory activity was evaluated in accordance of slightly revising the method described by *Winter et al.*[55]. Total 8 groups of six animals each are placed into individual cages.

- Group I: Control group animals received 10 ml/kg of 1% propylene glycol.
- Group II: Animals received standard Indomethacin at dose of 8 mg/kg i.p.
- Group III: Received AELS 100 mg/kg in 1% propylene glycol p.o.
- Group IV: Received AELS 200 mg/kg in 1% propylene glycol p.o.
- Group V: Received AELS 400 mg/kg in 1% propylene glycol p.o.
- Group VI: Received AEAR 100 mg/kg in 1% propylene glycol p.o.
- Group VII: Received AEAR 200 mg/kg in 1% propylene glycol p.o.
- Group VIII: Received AEAR 400 mg/kg in 1% propylene glycol p.o.

After 1 h of above drug treatment, 2% w/v Carrageenan solution (0.05 ml) were injected subcutaneously into sub plantar tissue of right hind paw of each animal and the contra lateral hind paws of same animal were also injected with 0.1 ml of saline as control. The increased paw volume was then plethysmographically measured at 0, 1, 2 and 3 h after injection of odematogenic agent. Actual edema volume was calculated from the difference between initial and subsequent reading and the percent inhibition activity was calculated by using the formula-

$$\%\;\mathrm{Inhibition}\;=\frac{\mathrm{Mean}\;\mathrm{paw}\;\mathrm{inflammation}\;\mathrm{of}\;\mathrm{control}\;-\mathrm{Mean}\;\mathrm{paw}\;\mathrm{inflammation}\;\mathrm{of}\;\mathrm{test}}{\mathrm{Mean}\;\mathrm{paw}\;\mathrm{inflammation}\;\mathrm{of}\;\mathrm{control}}\times 100$$

### Cotton-pellet granuloma model

Cotton pellet induced granuloma model in rats are used to produce sub-acute inflammation [71, 72]. Total 8 groups of six animals each are placed into individual cages. Sterilized dental cotton rolls (Johnson and Johnson, New Brunswick, NJ, USA) were cut into 5-mm pieces and two sterilized cotton pellets weighing 10 ± 1 mg were subcutaneously implanted along the flanks of axillae and groins bilaterally under ether anesthesia.

- Group I: Control group animals received 10 ml/kg of 1% propylene glycol.
- Group II: Animals received Standard Diclofenac sodium at dose of 40 mg/kg p.o.
- Group III: Received AELS 100 mg/kg in 1% propylene glycol p.o.
- Group IV: Received AELS 200 mg/kg in 1% propylene glycol p.o.
- Group V: Received AELS 400 mg/kg in 1% propylene glycol p.o.
- Group VI: Received AEAR 100 mg/kg in 1% propylene glycol p.o.
- Group VII: Received AEAR 200 mg/kg in 1% propylene glycol p.o.
- Group VIII: Received AEAR 400 mg/kg in 1% propylene glycol p.o.

Following drugs were administered orally for 6 consecutive days. On the 7^th^ day, the animals were sacrificed by cervical dislocation and the collected granulomas were dried in an oven for 24 hr at 60°C, then weighed and compared with control.

### Statistical analysis

All of values were expressed as mean ± SEM. Each results were analyzed for statistical significance using one-way ANOVA followed by Dunnet’s‘t’ test with *P < 0.05, **P < 0.01 and ***P < 0.001were considered as significant value.

## Abbreviations

AELS: Aqueous extract of *Launaea sarmentosa*
AEAR: Aqueous extract of *Aegialitis rotundifolia roxb*
COX: Cyclooxygenase
LOX: Lipoxygenase
NSAID: Non-steroidal anti-inflammatory drugs.
